# Physiological roles of pyruvate ferredoxin oxidoreductase and pyruvate formate-lyase in *Thermoanaerobacterium saccharolyticum* JW/SL-YS485

**DOI:** 10.1186/s13068-015-0304-1

**Published:** 2015-09-15

**Authors:** Jilai Zhou, Daniel G Olson, Anthony A Lanahan, Liang Tian, Sean Jean-Loup Murphy, Jonathan Lo, Lee R Lynd

**Affiliations:** Thayer School of Engineering, Hanover, NH 03755 USA; Department of Biological Sciences at Dartmouth College, Hanover, NH 03755 USA; BioEnergy Science Center, Oak Ridge, TN 37830 USA

**Keywords:** *Thermoanaerobacter saccharolyticum*, Pyruvate metabolism, Pyruvate ferredoxin oxidoreductase, Pyruvate formate-lyase, C1 metabolism

## Abstract

**Background:**

*Thermoanaerobacter saccharolyticum* is a thermophilic microorganism that has been engineered to produce ethanol at high titer (30–70 g/L) and greater than 90 % theoretical yield. However, few genes involved in pyruvate to ethanol production pathway have been unambiguously identified. In *T. saccharolyticum*, the products of six putative *pfor* gene clusters and one *pfl* gene may be responsible for the conversion of pyruvate to acetyl-CoA. To gain insights into the physiological roles of PFOR and PFL, we studied the effect of deletions of several genes thought to encode these activities.

**Results:**

It was found that pyruvate ferredoxin oxidoreductase enzyme (PFOR) is encoded by the *pforA* gene and plays a key role in pyruvate dissimilation. We further demonstrated that pyruvate formate-lyase activity (PFL) is encoded by the *pfl* gene. Although the *pfl* gene is normally expressed at low levels, it is crucial for biosynthesis in *T. saccharolyticum*. In *pforA* deletion strains, *pfl* expression increased and was able to partially compensate for the loss of PFOR activity. Deletion of both *pforA* and *pfl* resulted in a strain that required acetate and formate for growth and produced lactate as the primary fermentation product, achieving 88 % theoretical lactate yield.

**Conclusion:**

PFOR encoded by Tsac_0046 and PFL encoded by Tsac_0628 are only two routes for converting pyruvate to acetyl-CoA in *T. saccharolyticum*. The physiological role of PFOR is pyruvate dissimilation, whereas that of PFL is supplying C1 units for biosynthesis.

**Electronic supplementary material:**

The online version of this article (doi:10.1186/s13068-015-0304-1) contains supplementary material, which is available to authorized users.

## Background

**Fig. 1:**
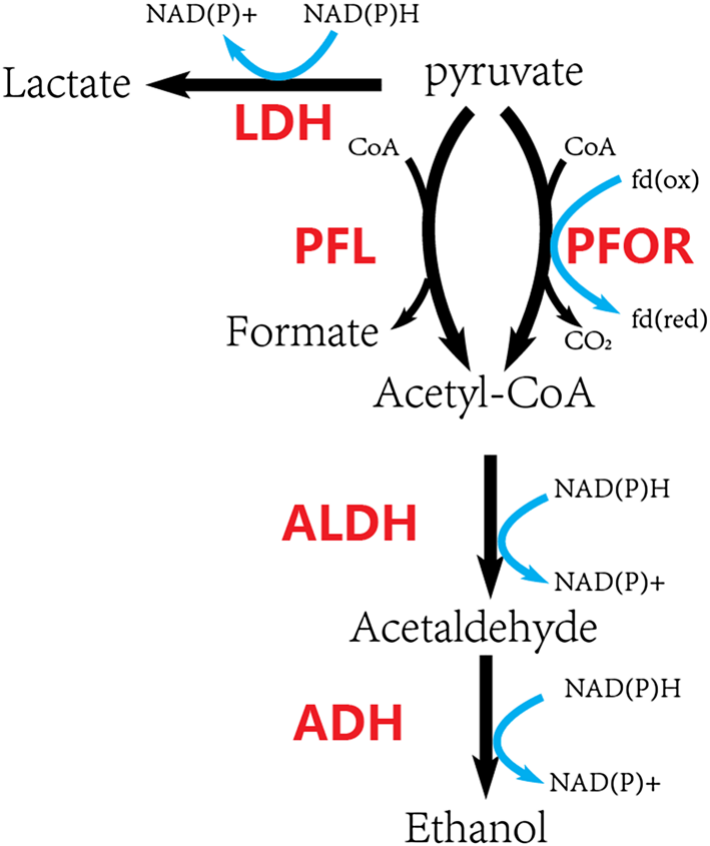
Metabolic pathway of pyruvate to ethanol in *T. saccharolyticum*. *Black arrows* represent the metabolic pathways; *blue arrows* represent the cofactor involved in the pathway. *LDH* lactate dehydrogenase, *PFL* pyruvate formate-lyase, *PFOR* pyruvate ferredoxin oxidoreductase, *ALDH* acetaldehyde dehydrogenase, *ADH* alcohol dehydrogenase. ALDH and ADH were thought to be catalyzed by bifunctional alcohol dehydrogenase in *T. saccharolyticum.*

*Thermoanaerobacterium saccharolyticum* is a thermophilic, anaerobic bacterium able to ferment hemicellulose, but not cellulose [1]. Wild-type strains produce ethanol, acetic acid and under some conditions lactic acid as the main products of fermentation, while engineered strains produce ethanol at high yield (>90 % of theoretical) and titer (30–70 g/L) [2–4]. Hemicellulose-utilizing thermophiles such as *T. saccharolyticum* commonly accompany cellulolytic microbes in natural environments [5] and are of interest as companion organisms for cellulolytic microbes such as *Clostridium thermocellum* in one-step, consolidated bioprocessing (CBP) without added enzymes [5, 6]. The pathway by which engineered strains of *T. saccharolyticum* produce ethanol is also of interest, because it is one of few examples of high-yield ethanol production thought to involve pyruvate conversion to acetyl-CoA via pyruvate ferredoxin oxidoreductase (PFOR) (Fig. 1), and because of the potential to engineer this metabolic pathway or important features thereof into other thermophiles [7]. Recently it has been shown that *adhE* is essential for ethanol production in *T. saccharolyticum* [8]; however, it is still unclear which genes are essential for pyruvate dissimilation.

The *T. saccharolyticum* genome includes six genes annotated as *pfor* and one as *pfl* (Table 1) [9–11]. Both genomic analysis and enzyme assays suggest that neither pyruvate dehydrogenase nor pyruvate decarboxylase is present in *T. saccharolyticum* [9]. *T. saccharolyticum* appears to have genes coding all three types of PFOR types defined by Chabriere et al. [12] based on quaternary structure. PFOR enzymes encoded by *pforA* and *pforC* are of the homodimer type, the *pforB* cluster codes for the heterodimer type, and PFORs encoded by cluster *pforD*, *pforE* and *pforF* appears likely to be of the heterotetramer type. The PFOR reaction is shown in Table 2, reaction A. There is disagreement in the literature about which genes are responsible for PFOR activity. Shaw et al. [9] identified Tsac_0380 and Tsac_0381 as the main *pfor* genes and detected methyl viologen-dependent PFOR activity in wild-type *T. saccharolyticum*. However, proteomic analysis indicates that PFOR encoded by *pforA* is the most abundant PFOR in glucose-grown cells [13]. The PFL reaction is shown by Table 2, equation C. Shaw et al. [9] identified Tsac_0628 as the gene encoding PFL enzyme. However, formate has not been detected as a product of fermentation in either the wild-type or the high ethanol-producing strain ALK2 [2].

**Table 1:** Clusters of *pfor* and *pfl* genes

Gene cluster	Gene	Annotated gene products^a^
*pforA*	Tsac_0046	Pyruvate ferredoxin/flavodoxin oxidoreductase
*pforB*	Tsac_0380	2-Oxoacid:acceptor oxidoreductase subunit alpha
	Tsac_0381	Pyruvate ferredoxin/flavodoxin oxidoreductase subunit beta
*pforC*	Tsac_0915	Pyruvate ferredoxin/flavodoxin oxidoreductase
	Tsac_1064	4Fe–4S ferredoxin
*pforD*	Tsac_1065	Pyruvate flavodoxin/ferredoxin oxidoreductase domain-containing protein
	Tsac_1066	Thiamine pyrophosphate TPP-binding domain-containing protein
	Tsac_1067	Pyruvate/ketoisovalerate oxidoreductase
	Tsac_2160	Pyruvate/ketoisovalerate oxidoreductase
*pforE*	Tsac_2161	Thiamine pyrophosphate TPP-binding domain-containing protein
	Tsac_2162	Pyruvate flavodoxin/ferredoxin oxidoreductase domain-containing protein
	Tsac_2163	4Fe–4S ferredoxin
	Tsac_2177	Pyruvate/ketoisovalerate oxidoreductase subunit gamma
*pforF*	Tsac_2178	Thiamine pyrophosphate TPP-binding domain-containing protein
	Tsac_2179	Pyruvate flavodoxin/ferredoxin oxidoreductase domain-containing protein
	Tsac_2180	4Fe–4S ferredoxin
*pfl*	Tsac_0628	Pyruvate formate-lyase
	Tsac_0629	Pyruvate formate-lyase-activating enzyme

^a^The gene product annotations were based on NCBI genome project (NC_017992.1).

**Table 2:** Potential reactions related to pyruvate dissimilation in *T. saccharolyticum*

Reaction ID	Enzyme name	Reaction catalyzed by the enzyme
A	Pyruvate ferredoxin oxidoreductase (PFOR)	Pyruvate + CoA + ferredoxin (ox) → acetyl-CoA + ferredoxin (red) + CO_2_
B	Ferredoxin/NAD(P)H oxidoreductase (FNOR)	Ferredoxin (red) + NAD(P)^+^ + H^+^ → ferredoxin (ox) + NAD(P)H
C	Pyruvate formate-lyase (PFL)	Pyruvate + CoA → acetyl-CoA + formate
D	Formate dehydrogenase (FDH)	Formate + NAD(P)^+^ → CO_2_ + NAD(P)H

To achieve high-yield ethanol production in fermentative microbes with catabolism featuring pyruvate conversion to acetyl-CoA, the electrons from this oxidation must end up in ethanol, presumably via nicotinamide cofactors. In the case of PFOR, this means that electrons from reduced ferredoxin need to be transferred to NAD^+^ or NADP^+^. In the case of PFL, this means that electrons from formate must be transferred to NAD^+^ or NADP^+^. Shaw et al. [9] have detected ferredoxin-NAD(P)H activity, corresponding to reaction B in Table 2, in cell extracts. A *fnor* gene (Tsac_2085) has also been identified [9]. A recent study has confirmed that the electron-bifurcating enzyme complex NfnAB, encoded by Tsac_2085 and Tsac_2086, plays a key role for generating NADPH from reduced ferredoxin in *T. saccharolyticum* [14]. Formate dehydrogenase (FDH) is another possible route for electron transfer to ethanol. However FDH (equation D in Table 2) has not been found in *T. saccharolyticum*, either by sequence homology or enzyme assay [9–11].

The conversion of pyruvate to acetyl-CoA is thought to proceed by the PFOR reaction in *T. saccharolyticum*; however, few of the specific genes responsible for ethanol formation from pyruvate in *T. saccharolyticum* have been unambiguously identified. For example, in the closely related species, *C. thermocellum*, despite the presence of a complete genome sequence, gene deletions and enzyme assays were required to determine a number of key aspects of central metabolism [15]. Following this, we decided to closely examine pyruvate metabolism in *T. saccharolyticum*. In particular, we wished to confirm whether PFOR is responsible for pyruvate dissimilation, identify which of the many PFOR enzymes are most important, gain insight into the function of PFL and examine the physiological consequences of deleting these genes individually and in combination.

## Results

### Deletion of *pfor*

**Fig. 2:**
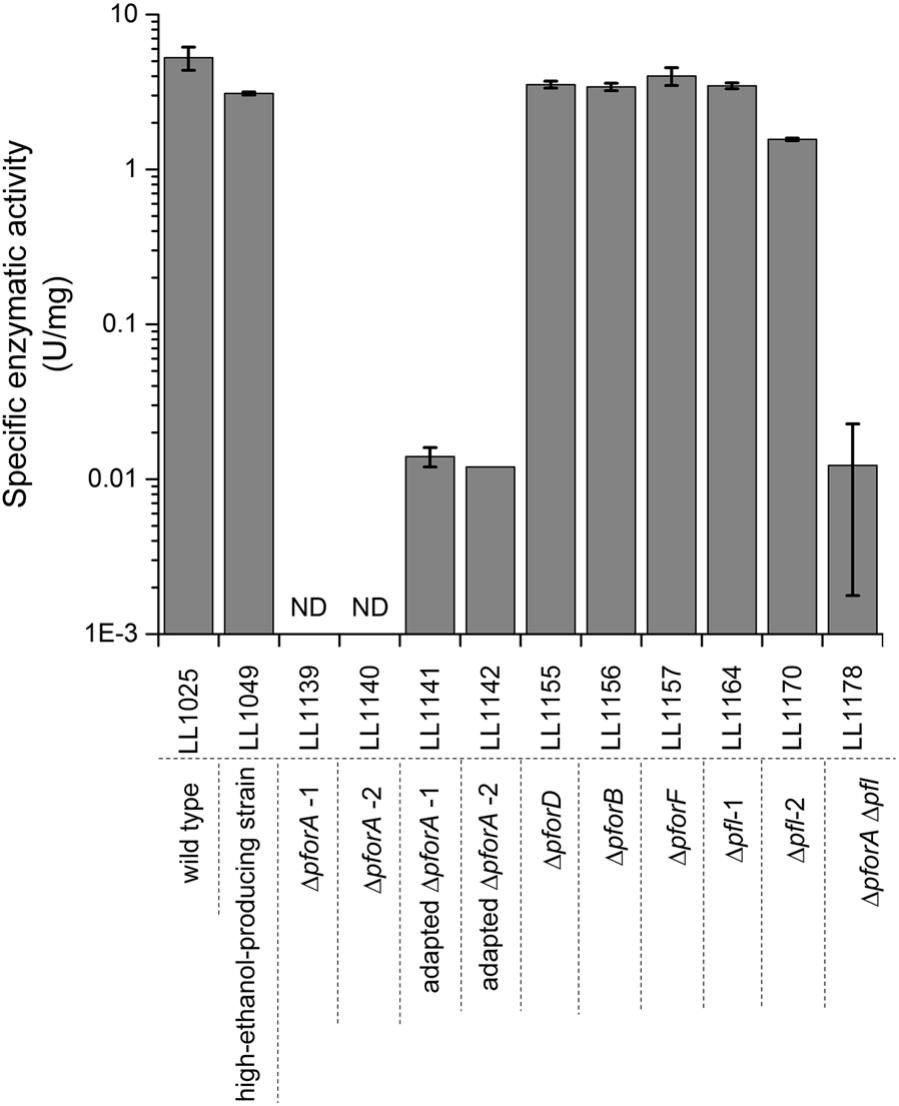
Enzymatic activity of pyruvate ferredoxin oxidoreductase from cell-free extract of *T. saccharolyticum* mutants. *Error bars* represent the standard deviation of three replicates. ND (not detected), the specific activities were below detection limit 0.005 U/mg.

There are six gene clusters in the *T. saccharolyticum* genome annotated as pyruvate ferredoxin/flavodoxin oxidoreductases according to KEGG [10, 11] (Table 1). In our first round of deletions, we succeeded in deleting four of the six clusters: *pforA*, *pforB*, *pforD* and *pforF* separately in the wild-type strain (LL1025). Deletion of *pforA* resulted in the elimination of PFOR enzyme activity. The other deletions did not affect PFOR activity (Fig. 2). As expected from the enzyme assay data, only the *pforA* deletion resulted in a change in products of fermentation (Table 3).

**Table 3:** Fermentation profiles of *T. saccharolyticum* knockout strains

Strains		Additions to medium	Fermentation profile^a^ Unit: mmol in 50 mL culture												
Name	Description		Consumed cellobiose	Residual cellobiose	Formate	Lactate	Acetate	Ethanol	Succinate	Pyruvate	Malate	Pellet carbon	Hydrogen	Carbon recovery (%)	Electron recovery (%)
LL1025	Wild type	None	0.70	0.00	0.01	0.28	0.71	1.18	0.01	0.00	0.00	0.80	1.68	90	94
LL1049	Ethanologenic strain	None	0.70	0.00	0.22	0.00	0.04	2.10	0.00	0.00	0.04	0.72	0.22	86	90
LL1139	LL1025 Δ*pforA*-1	None	0.07	0.63	0.15	0.04	0.05	0.09	0.00	0.00	0.00	0.18	0.01	98	99
LL1140	LL1025 Δ*pforA*‏-2	None	0.02	0.68	0.01	0.00	0.00	0.00	0.00	0.00	0.00	0.19	0.10	100	101
LL1141	Adapted from LL1139	None	0.37	0.34	0.28	0.67	0.06	0.29	0.00	0.06	0.03	0.27	0.02	91	92
LL1142	Adapted from LL1140	None	0.34	0.36	0.38	0.26	0.03	0.42	0.02	0.22	0.03	0.21	0.04	89	90
LL1155	LL1025 Δ*pforD*	None	0.70	0.00	0.02	0.39	0.72	1.20	0.01	0.00	0.00	0.72	1.65	91	94
LL1156	LL1025 Δ*pforB*	None	0.70	0.00	0.02	0.15	0.80	1.24	0.01	0.00	0.00	0.89	1.74	92	94
LL1157	LL1025 Δ*pforF*	None	0.70	0.00	0.00	0.38	0.76	1.19	0.00	0.00	0.00	0.73	1.64	91	94
LL1159	LL1049 Δ*pforA*	None	0.07	0.63	0.01	0.00	0.00	0.01	0.00	0.00	0.00	0.23	0.05	92	92
LL1164	LL1025 Δ*pfl*–1	Formate	0.48	0.21	−0.02^b^	1.13	0.19	0.33	0.00	0.00	0.00	0.47	0.48	96	98
LL1170	LL1025 Δ*pf*-2	Formate	0.69	0.00	−0.03^b^	0.24	0.81	1.22	0.00	0.00	0.00	0.92	0.69	92	95
LL1178	LL1025 Δ*pforA*; Δ*pfl*	Formate and Acetate	0.48	0.25	−0.01^c^	1.69	−0.12^c^	0.08	0.00	0.00	0.01	0.31	0.00	94	96

The amount of fermentation end products are reported in millimoles in a volume of 50 mL serum bottle. The amounts of Initial cellobiose were 0.70 mmol for all fermentations. Cultures were incubated for 72 h at 55 °C with an initial pH of 6.2 in MTC-6 medium.

^a^The standard deviations were less than 10 % for cellobiose, formate, lactate, acetate, ethanol, pyruvate, succinate and malate, which were measured by HPLC. For pellet carbon and hydrogen measurement, the standard deviation was less than 2 %. The calculated carbon recovery and electron recovery have a combined standard deviation less than 5 %.

^b^To improve the growth of LL1164, LL1170, 0.20 mmol formate was added into 50 mL MTC-6 medium. Negative values represent that a certain amount of sodium formate was consumed during fermentation.

^c^LL1178 requires supplementation of both formate and acetate to grow in MTC-6 medium. 0.20 mmol sodium formate and 0.20 mmol sodium acetate were added into 50 mL MTC-6 medium. Negative values represent that a certain amount of sodium formate and sodium acetate was consumed during fermentation.

**Fig. 3:**
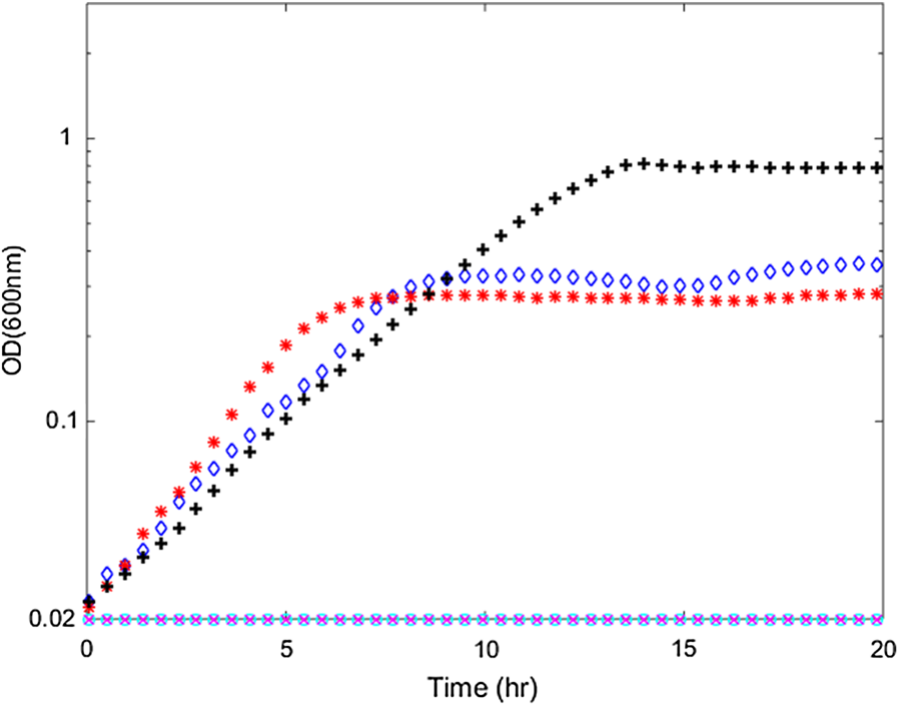
Growth curves of Δ*pfor* strains in MTC-6 medium. *Black plus signs* represent wild-type strain (LL1025), *cyan circles* represent Δ*pfor*-1, *magenta crosses* represent Δ*pfor*-2, *blue diamonds* represent adapted Δ*pfor*-1, *red stars* represent adapted Δ*pfor*-2.

Fermentation profiles for individual colonies of the *pforA* deletion strain revealed two different phenotypes with respect to lactate production. These strains were named LL1139 and LL1140. Strain LL1140 had less lactate production than LL1139 (Table 3). Both LL1139 and LL1140 showed elevated formate production compared to the wild-type strain. They were not able to consume more than 10 % of the 5 g/L cellobiose initially present in the medium (Table 3). The maximum ODs of these two strains in MTC-6 medium were reduced by 60 % in the presence of yeast extract (Additional file 1: Figure S1) and over 90 % in the absence of yeast extract (Fig. 3). Growth rates and lag phases of these two strains were similar to wild type with the presence of yeast extract (Additional file 1: Figure S1), but they did not grow without yeast extract in the MTC-6 medium over the course of 20 h (Fig. 3). Of the eight colonies analyzed, seven had the LL1139 phenotype and only one had the LL1140 phenotype.

To improve strain fitness, we adapted both LL1139 and LL1140 in MTC-6 medium for 20 transfers (approximately 140 generations) until no additional changes in growth rate were observed. Adapted cultures of strains LL1139 and LL1140 were named LL1141 and LL1142, respectively. Both strains produced more formate compared with their unadapted parent strains. Strain LL1141 produced more lactate and less pyruvate than LL1142, but otherwise their fermentation profiles were similar. Both strains were able to consume about half of the 5 g/L cellobiose initially present in the medium (Table 3), and the maximum cell density and growth rate were greater than the unadapted parent strains in the defined medium but did not recover to wild-type level (Fig. 3).

**Fig. 4:**
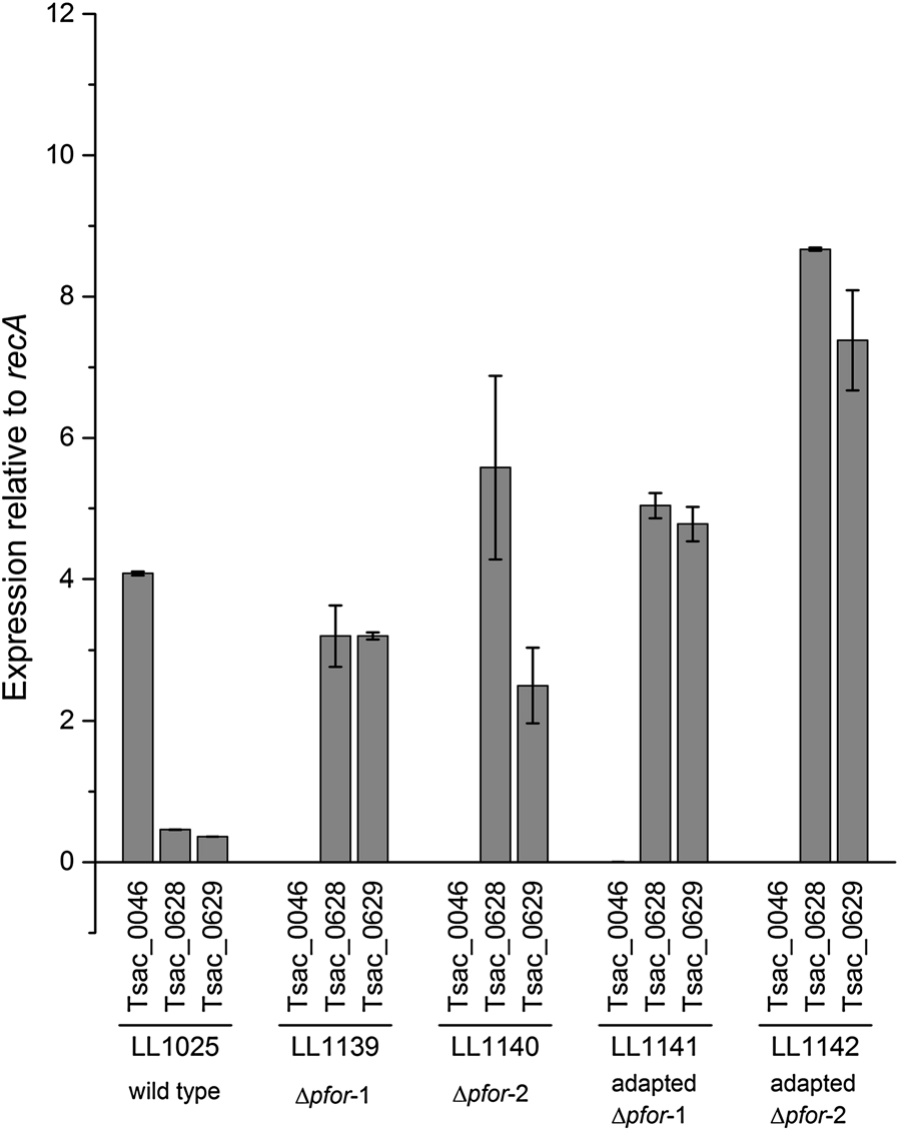
Relative mRNA level of Tsac_0046, Tsac_0628 and Tsac_0629 in adapted *pforA* deletion strains. Tsac_0046 encodes pyruvate ferredoxin oxidoreductase, Tsac_0628 encodes pyruvate formate-lyase and Tsac_0629 encodes pyruvate formate-lyase activating enzyme. The *recA* gene is Tsac_1846, annotated as a DNA recombination and repair protein, which usually has a consistent expression level across many strains and environmental conditions.

In all *pfor* deletion strains, the expression levels of pyruvate formate-lyase genes were increased about sixfold compared with the parent strain (Fig. 4). Transcriptional analysis also indicated that *pfl* had higher expression level in LL1142 than LL1141, which corresponds to higher formate production in LL1142.

We also deleted *pforA* in the high ethanol-producing strain of *T. saccharolyticum*, LL1049, previously developed by Mascoma [4]. The resulting strain was named LL1159. This strain grew slower than LL1139 or LL1140 in MTC-6 medium and it was unable to consume more than 10 % of 5 g/L cellobiose (Table 3).

### Deletion of *pfl*

To investigate the physiological role of PFL in *T. saccharolyticum*, we deleted the *pfl* gene cluster in the wild type (LL1025). The *pfl* deletion in strain LL1025 gave two different phenotypes (high lactate and low lactate), which were stored as strain LL1164 and LL1170. Of eight colonies picked, two had the LL1164 phenotype and six had the LL1170 phenotype. Strain LL1170 consumed more cellobiose, produced more acetate and ethanol and less lactate than strain LL1164 (Table 3).

**Fig. 5:**
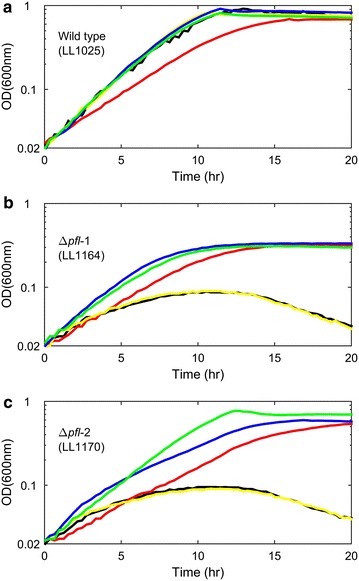
Growth of *pfl* deletion strains in MTC-6 medium (*black*), and with 4 mM formate (*red*), with 4 mM glycine (*yellow*), MTC with 4 mM serine (*blue*), MTC with 10 mg/L lipoic acid (*green*).

Both *pfl* deletion strains grew more poorly in MTC-6 medium than in CTFUD medium. The biggest difference between CTFUD and MTC-6 medium is the presence of yeast extract. The addition of yeast extract could restore the growth of *pfl* deletion strains in the MTC-6 medium (Additional file 2: Figure S2). The growth of both strains was stimulated by addition of formate, serine or lipoic acid (Fig. 5). In cases where formate was added, a small amount was consumed by all three strains (less than 1 mM, which is equivalent to 0.05 mmol in 50 mL culture as shown in Table 3).

### Double deletion of *pfor* and *pfl*

In the adapted *pforA* deletion strains (LL1141 and LL1142), formate production was significantly increased, and carbon flux toward acetate and ethanol formation was presumptively via the PFL reaction. To show that PFOR encoded by *pforA* and PFL encoded by *pfl* were the only two routes for the conversion of pyruvate to ethanol in *T. saccharolyticum*, we deleted *pfl* in strain LL1141 (which already contained the *pforA* deletion). To create this deletion, it was necessary to supplement the medium with 4 mM sodium acetate.

The resulting *pfor*/*pfl* double deletion strain (LL1178) consumed about 70 % of the 5 g/L cellobiose initially present, which was about the same as its parent strain (LL1141). It required sodium acetate for growth, even in the presence of yeast extract. Lactate became the main product of fermentation, with 3.5 mol of lactate produced for each mole of cellobiose consumed (or 88 % of the theoretical maximum yield) (Table 3).

### Genomic sequence of mutants

Comparing resequencing results for the *pfor* deletion strains (LL1139 and LL1140) (Additional file 3: Table S1), we found a mutation in lactate dehydrogenase gene of LL1140, which was maintained during the adaptation process and also found in strain LL1142 (adapted version of LL1140).

As described before, we isolated two different phenotypes (high and low lactate) when we deleted *pfl* in *T. saccharolyticum*. They were named as LL1164 and LL1170, respectively. Strain LL1164 could not consume all 5 g/L cellobiose initially present in the medium and produced lactate as the main product of fermentation. After comparing the genome resequencing data of LL1164 and LL1170 (two phenotypes of *pfl* deletion strains from wild-type *T. saccharolyticum*), we found two mutations that were present in LL1164, but not in LL1170. One mutation is a synonymous mutation in Tsac_1304, which is annotated as uncharacterized protein, the other one is found in Tsac_1553, which is annotated as ferredoxin hydrogenase.

## Discussion

### The major route for pyruvate dissimilation

Wild-type *T. saccharolyticum* produces 2.7 mol of C2 products (ethanol and acetate) for each mole of cellobiose consumed (since the theoretical maximum is 4, this is 68 % of the theoretical maximum yield). Deletion of the primary *pfor* gene, *pforA*, resulted in a dramatic decrease in growth, indicating the importance of *pforA* in pyruvate dissimilation. Since ethanol was still produced, it was hypothesized that *pfl* partially compensated for the deletion of *pfor*. Creation of a double deletion strain (LL1178), with both *pfor* and *pfl* deleted, produced almost no C2 products and carbon flux was redirected to lactate production. The C2 yield in this strain is −0.08 mol per mole of cellobiose consumed (the slight negative value is due to the consumption of sodium acetate), whereas the C3 (i.e., lactate) yield is 3.52 (88 % of theoretical).

After the deletion of Δ*pforA*, the mRNA level of *pfl* increased in all strains. We did not find any consistent mutation in all Δ*pforA* strains that led to such an increase. It is possible that the transcription is upregulated by some intermediate metabolites that may accumulate in these strains, such as pyruvate, but we do not have any direct evidence for this. In adapted *pforA* deletion strains (LL1141 and LL1142), the flux through PFL was increased, which was observed by increased production of formate. If C2 products were produced exclusively via the PFL pathway, formate production and C2 yield should be equivalent on a molar basis. For strain LL1141, formate production can account for about 80 % of the C2 products. For strain LL1142, formate production can account for about 84 % of the C2 products (Table 3). One possible explanation for the residual C2 production is consumption of formate for biosynthesis. Another possible explanation is PFOR activity from a gene cluster other than *pforA*. Although PFOR activity was eliminated after deletion of *pforA*, adaptation resulted in the appearance of very low levels of PFOR activity (less than 1/100th) that could be from one of the other annotated *pfor* genes (Fig. 2).

### The gene encoding PFOR

Based on data from enzyme assay and gene deletions, it appears that *pforA* is the gene encoding the primary PFOR enzyme in *T. saccharolyticum*, which is different from the gene cluster, *pforB*, as suggested by Shaw et al. [9]. Single deletion of the *pforA* cluster in wild-type *T. saccharolyticum* completely eliminated the PFOR activity, while deletions of other *pfor* gene clusters had no effect under tested conditions (Fig. 2). This result is also consistent with proteomic data for *T. saccharolyticum*, in which PFOR encoded by *pforA* is the most abundant protein among all PFOR enzymes [13]. Enzymes encoded by other *pfor* gene clusters are expressed at a much lower level, at least ten times lower than that encoded by *pforA* [13]. The role of these other gene clusters remains unknown.

### Pyruvate dehydrogenase and pyruvate decarboxylase activity

The genes for PDC and PDH were absent in the genome of *T. saccharolyticum* [9–11]. Shaw et al. also did not detect PDH or PDC activities by enzyme assay (which we have confirmed). There are reports that PFOR can decarboxylate pyruvate directly to acetaldehyde, functioning as pyruvate decarboxylase (PDC) in *Pyrococcus furiosus* [16] and *Thermococcus guaymasensis* [17]. Although in both cases, the acetyl-CoA production rates are higher than acetaldehyde production rates (roughly 5:1 in both organisms [17]), the PDC side activity of PFOR is still thought to be one of the options for acetaldehyde production in hyperthermophiles [17]. Another possibility is through aldehyde ferredoxin oxidoreductase (AOR), which can convert acetate to acetaldehyde [18, 19]. According to this ratio of PFOR activity versus PDC activity, the PDC activity should be in the order of 0.1–1 U/mg if the PFOR in *T. saccharolyticum* has this side activity. However we did not detect PDC activity in cell extracts (<0.005 U/mg), so this activity (if it exists) does not likely play a significant physiological role.

We also examined the existence of PDH in several other species that are closely related to *T. saccharolyticum* (Table 4). In some *Thermoanaerobacter* species, they possess all genes required to encode the PDH complex, but their function and physiological roles remain to be determined experimentally.

**Table 4:** Comparison of genes involved in pyruvate metabolism and C1 metabolism between *T. saccharolyticum* and its relative species

Organisms	Enzymes					
	PFOR	PDH	PFL	Glycine cleavage system	Lipoic acid synthesis	Lipoic salvage system
*Clostridium thermocellum* DSM1313	+	−	+	−	−^b^	−^b^
*Clostridium clariflavum* DSM 19732	+	−	+	−	−^b^	−^b^
*Clostridium stercorarium* subsp. *stercorarium* DSM 8532	+	+	−	+	+	−
*Thermoanaerobacter saccharolyticum* JW/SL-YS485	+	−	+	+	−	+
*Thermoanaerobacter tengcongensis* MB4(T)	+	+	−	+	+	+
*Thermoanaerobacter* sp. X514	+	+	−	+	+	+
*Thermoanaerobacter pseudethanolicus* ATCC 33223	+	+	−	+	+	+
*Thermoanaerobacter italicus* Ab9	+	−	−	+	+	+
*Thermoanaerobacter mathranii* subsp. *mathranii* A3	+	−	−	+	+	+
*Thermoanaerobacter brockii subsp. finnii* Ako-1	+	+	−	+	+	+
*Thermoanaerobacter wiegelii* Rt8.B1	+	+	−	+	+	+
*Thermoanaerobacter kivui* DSM 2030	+	−	−	+	+	+
*Thermoanaerobacterium thermosaccharolyticum* DSM571	+	−	+^a^	+	−	+
*Thermoanaerobacterium xylanolyticum* LX-11	+	−	+	+	−	+
*Caldicellulosiruptor saccharolyticus* DSM 8903	+	−	−	−	+	−
*Caldicellulosiruptor bescii* DSM 6725	+	−	−	−	+	−
*Caldicellulosiruptor obsidiansis* OB47	+	−	−	−	+	−
*Caldicellulosiruptor hydrothermalis* 108	+	−	−	−	+	−
*Caldicellulosiruptor owensensis* OL	+	−	−	−	+	−
*Caldicellulosiruptor kristjanssonii* 177R1B	+	−	−	−	−^b^	−^b^
*Caldicellulosiruptor kronotskyensis* 2002	+	−	−	−	+	−
*Caldicellulosiruptor lactoaceticus* 6A	+	−	−	−	−^b^	−^b^

^a^ *T. thermosaccharolyticum* DSM571 has *pfl* annotated, whereas *T. thermosaccharolyticum* M0795 does not have it. It is also confirmed with protein blast using PFL protein sequence from *T. saccharolyticum.*

^b^No information about lipoic acid metabolism of *C. thermocellum* DSM1313, *C. clariflavum* DSM 19732, *C. kristjanssonii* 177R1B and *C. lactoaceticus* 6A in KEGG. The existence of lipoic acid biosynthesis and lipoic salvage system are confirmed by protein blast using lipoyl synthase from *C. bescii* and lipoate protein ligase from *T. saccharolyticum.*

### Role of *pfl* and C1 metabolism

Pyruvate formate-lyase was only expressed at low levels and was not the major route for pyruvate dissimilation in the wild-type strain. It was, however, required for growth of *T. saccharolyticum* grown in MTC-6 medium. The consumption of added formate and restoration of stronger growth upon addition of formate by all *pfl* deletions strains (Table 3) supports the hypothesis that PFL is required for biosynthesis.

It has been previously reported that PFL has an anabolic function in *Clostridium* species and furnishes cells with C1 units [20, 21]. The results presented here suggest that this might also be the case in *T. saccharolyticum*, which belongs to class *Clostridia*. In *Clostridium acetobutylicum*, 13C labeling experiments showed that over 90 % of C1 units in biosynthetic pathways come from the carboxylic group of pyruvate and are likely to be derived from the PFL reaction [22]. Due to the impaired growth of *pfl* deletion strains, we think this is likely the case in *T. saccharolyticum* also. In the case of *C. acetobulyticum*, Amador-Noguez et al. [22] found that glycine is not formed from serine, and thus that the methyl group from serine is not transferred to tetrahydrofolate (THF) in this organism. However, in the case of *T. saccharolyticum*, the growth of *pfl* deletion strains was restored by the addition of serine, suggesting that C1 units are transferred from serine to THF.

**Fig. 6:**
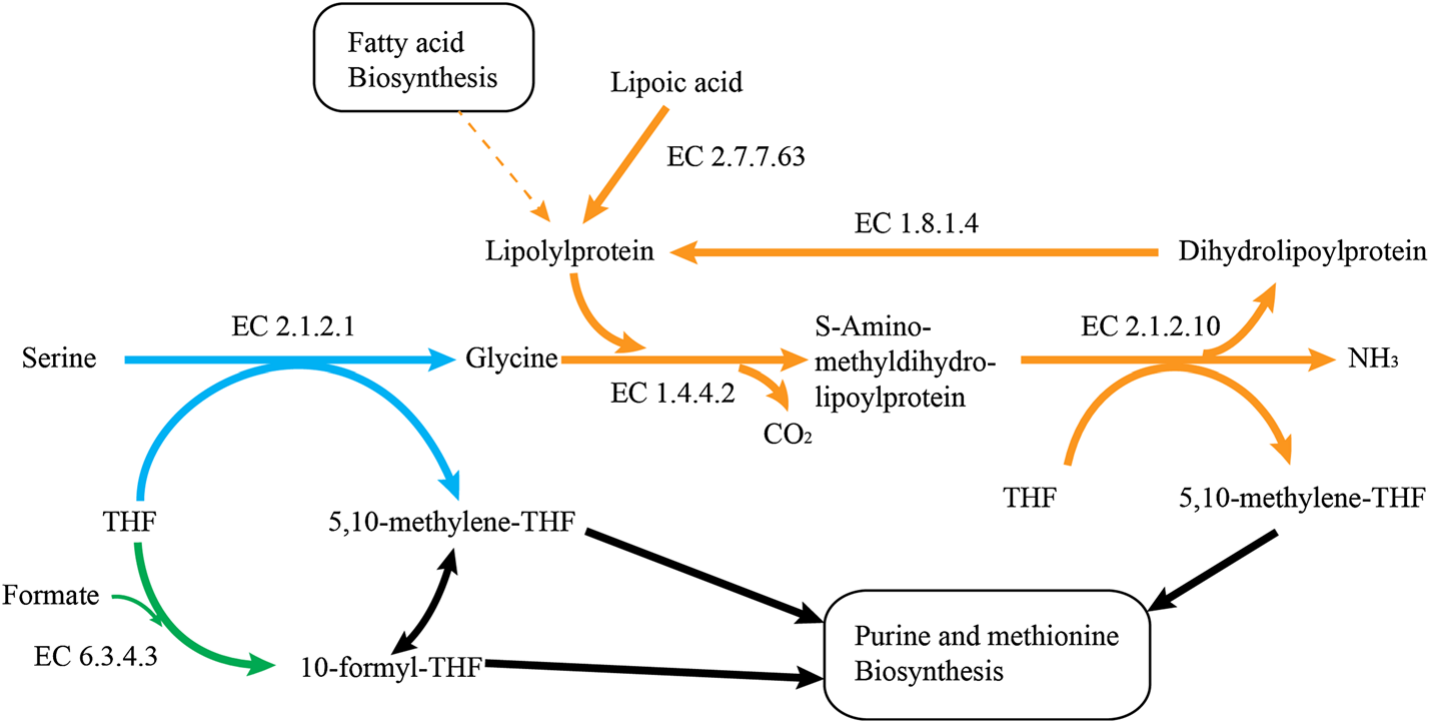
Proposed one carbon metabolic pathway in *T. saccharolyticum*. *Green arrows* indicate the pathway for 10-formyl-THF production. Note that this pathway requires formate, which is presumably generated by PFL in *T. saccharolyticum*. *Blue arrows* indicate the active pathways of *pfl* deletion strains grown in MTC-6 supplemented with additional serine. *Orange arrows* indicate active pathways in *pfl* deletion strains grown in MTC-6 supplemented with additional lipoic acid. *EC numbers* represent enzymes responsible for catalyzing that reaction. In *T. saccharolyticum*, formate tetrahydrofolate ligase (EC 6.3.4.3) is encoded by Tsac_0941.

Although additional glycine did not stimulate the growth of *T. saccharolyticum*, additional lipoic acid did improve growth (Fig. 5). In fact, *T. saccharolyticum* has all of the genes required for the glycine cleavage system and the lipoic acid salvage system. Since it does not have lipoic acid biosynthesis pathways, it required additional lipoic acid for H protein formation, which is essential for the glycine cleavage system [23]. The proposed one carbon metabolism in *T. saccharolyticum* is shown in Fig. 6 based on the generic C1 metabolism network from KEGG [10].

Among other species that we have examined, most of the *Thermoanaerobacter* species have the glycine cleavage system and either the lipoic acid biosynthesis or the lipoic acid salvage system for H protein formation (Table 4). However, *Caldicellulosiruptor* species do not have either PFL or glycine cleavage system. Therefore, we think they may use serine aldolase (EC 2.1.2.1) for the supply of C1 units.

### Mutations found in genomic resequencing

In one *pfor* deletion strain lineage (lineage 2, Additional file 3: Table S1), which includes Δ*pforA*-2 (strain LL1140) and its adapted descendant (strain LL1142), we found an SNP in lactate dehydrogenase (Tsac_0179). This SNP causes an amino acid change from asparagine to serine. According to the protein structure of LDH from *Bacillus stearothermophilus* [24], which shares 48 % identity with that from *T. saccharolyticum*, we found this mutation was near the catalytic site. We suspect that this SNP may explain the decrease in lactate production in strains LL1140 and LL1142.

In one *pfl* deletion strain (LL1164) but not another (strain LL1170, a different colony from the *pfl* deletion experiment, see previous description), an SNP was found in the ferredoxin hydrogenase, subunit B (*hfsB*, Tsac_1153). A non-functional *hfs* gene could inhibit the PFOR reaction by preventing the oxidation of reduced ferredoxin. Shaw et al. [25] found that deletion of the entire *hfs* operon resulted in a decrease in hydrogen and acetate production and increase in lactate production. We see similar trends for hydrogen, acetate and lactate. Shaw et al. found a slight decrease in ethanol production (22 %), whereas we see a much larger decrease (73 %). The similarities in the patterns of fermentation data between our *hfsB* mutant and the *hfs* deletion from Shaw et al. suggest that the *hfs* mutation may in fact be responsible for the change in distribution of products of fermentation between strains LL1164 and LL1170.

## Conclusion

In this study, we have identified genes and enzymes responsible for pyruvate ferredoxin oxidoreductase and pyruvate formate-lyase activities in *T. saccharolyticum*. The primary physiological role of PFOR appears to be pyruvate dissimilation, while the role of PFL appears to be supplying C1 units in biosynthesis. PFOR encoded by Tsac_0046 and PFL encoded by Tsac_0628 are only two routes for converting pyruvate to acetyl-CoA in *T. saccharolyticum*. The combination deletion of these two genes virtually eliminated pyruvate flux to acetyl-CoA, which can be seen by the shift of carbon flux to lactate production at high yield (88 % of theoretical).

## Methods

### Strains and plasmids

*T. saccharolyticum* JW/SL-YS485 (aka LL1025, DSM8691) was kindly provided by Juergen Wiegel (University of Georgia, Athens, GA, USA) and stored in the laboratory strain collection. Strain LL1049 (aka M1442) was a gift from the Mascoma Corporation [4]. All other strains were from commercial sources or developed in our laboratory (Table 5). Plasmids are described in Table 5.

**Table 5:** Strains and plasmids

Strain or plasmid	Description	Accession number	References
Strains			
*E. coli*			
DH5α	*E. coli* cloning strains	N/A	New England Biolabs
*T. saccharolyticum*			
LL1025	Wild-type strain	SRA234880	[32]
LL1040 (aka ALK2)	High ethanol-producing strain, Kan^r^, Erm^r^	N/A	[2]
LL1049 (aka M1442)	High ethanol-producing strain	SRA233073	[4]
LL1139	LL1025 Δ*pforA* ::Kan^r^, colony 1	SRA234882	This study
LL1140	LL1025 Δ*pforA* ::Kan^r^, colony 2	SRA233066	This study
LL1141	Adapted LL1139	SRA234883	This study
LL1142	Adapted LL1140	SRA234884	This study
LL1155	LL1025 Δ*pforD* ::‏ Kan^r^,	N/A	This study
LL1156	LL1025 Δ*pforB* :: ‏Kan^r^,	N/A	This study
LL1157	LL1025 Δ*pforF* :: ‏Kan^r^,	N/A	This study
LL1159	LL1049 Δ*pforA* :: ‏Kan^r^,	N/A	This study
LL1164	LL1025 Δ*pfl* :: Kan^r^, colony 1	SRA233080	This study
LL1170	LL1025 Δ*pfl* :: Kan^r^, colony 2	SRA233074	This study
LL1178	LL1141 Δ*pfl* :: Erm^r^	SRA234885	This study
Plasmids			
pMU433	Cloning vector *pta/ack*, Kan^r^		[33]
pZJ13	*pforA* knockout, Kan^r^, *pta/ack*	KP057684	This study
pZJ15	*pforB* knockout, Kan^r^, *pta/ack*	KP057685	This study
pZJ16	*pforD* knockout, Kan^r^, *pta/ack*	KP057686	This study
pZJ17	*pforF* knockout, Kan^r^, *pta/ack*	KP057687	This study
pZJ20	*pfl* knockout, Kan^r^, *pta/ack*	KP057688	This study
pZJ23	Cloning vector Erm^r^, Amp^r^	KP057689	This study
pZJ25	*pfl* knockout, Erm^r^, Amp^r^	KP057690	This study

Note, accession numbers for strains refer to raw resequencing data from the JGI Sequence Read Archive. Accession numbers for plasmids refer to the Genbank database.

*Kanr* kanamycin resistant, *Ermr* erythromycin resistant, *Ampr* ampicillin resistant, *pta/ack* is a negative selective marker.

### Media and growth conditions

Genetic modifications of *T. saccharolyticum* JW/SL-YS485 strains were performed in CTFUD medium, containing 1.3 g/L (NH_4_)_2_SO_4_, 1.5 g/L KH_2_PO_4_, 0.13 g/L CaCl_2_·2H_2_O, 2.6 g/L MgCl_2_·6H_2_O, 0.001 g/L FeSO_4_·7H_*2*_O, 4.5 g/L yeast extract, 5 g/L cellobiose, 3 g/L sodium citrate tribasic dihydrate, 0.5 g/L l-cysteine-HCl monohydrate, 0.002 g/L resazurin and 10 g/L agarose (for solid media only). The pH was adjusted to 6.7 for selection with kanamycin (200 μg/mL), or adjusted to 6.1 for selection with erythromycin (25 μg/mL).

Measurement of fermentation products and growth of *T. saccharolyticum* were performed in MTC-6 medium [26], including 5 g/L cellobiose, 9.25 g/L MOPS (morpholinepropanesulfonic acid) sodium salt, 2 g/L ammonium chloride, 2 g/L potassium citrate monohydrate, 1.25 g/L citric acid monohydrate, 1 g/L Na_2_SO_4_, 1 g/L KH_2_PO_4_, 2.5 g/L NaHCO_3_, 2 g/L urea, 1 g/L MgCl_2_·6H_2_O, 0.2 g/L CaCl_2_·H_2_O, 0.1 g/L FeCl_2_·6H_2_O,1 g/L l-cysteine HCl monohydrate, 0.02 g/L pyridoxamine HCl, 0.004 g/L *p*-aminobenzoic acid (PABA), 0.004 g/L D-biotin, 0.002 g/L vitamin B12, 0.04 g/L thiamine, 0.005 g/L MnCl_2_·4H_2_O, 0.005 g/L CoCl_2_·6H_2_O, 0.002 g/L ZnCl_2_, 0.001 g/L CuCl_2_·2H_2_O, 0.001 g/L H_3_BO_3_, 0.001 g/L Na_2_MoO_4_·2H_2_O and 0.001 g/L NiCl_2_·6H_2_O. It was prepared by combining six sterile solutions (A–F) with minor modification under nitrogen atmosphere as described before [15]. All six solutions were sterilized through a 0.22 μm filter (Corning, #430517). Solution A, concentrated 2.5-fold, contained cellobiose, MOPS sodium salt and distilled water. Solution B, concentrated 25-fold, contained potassium citrate monohydrate, citric acid monohydrate, Na_2_SO_4_, KH_2_PO_4_, NaHCO_3_ and distilled water. Solution C, concentrated 50-fold, contained ammonium chloride and distilled water. Solution D, concentrated 50-fold, contained MgCl_2_·6H_2_O, CaCl_2_·H_2_O, FeCl_2_·6H_2_O and l-cysteine HCl monohydrate. Solution E, concentrated 50-fold, contained thiamine, pyridoxamine HCl, *p*-aminobenzoic acid (PABA), D-biotin and vitamin B12. Solution F, concentrated 1000-fold, contained MnCl_2_·4H_2_O, CoCl_2_·6H_2_O, ZnCl_2_, CuCl_2_·2H_2_O, H_3_BO_3_, Na_2_MoO_4_·2H_2_O and NiCl_2_·6H_2_O. Some fermentations required supplementation with additional components. These were added after the first six solutions were combined. The final pH was adjusted to 6.1. Fermentations of *T. saccharolyticum* were done in 125-mL glass bottles at 55 °C under a nitrogen atmosphere. The working volume was 50 mL with shaking at 250 rpm. Fermentations were allowed to proceed for 72 h, at which point samples were collected for analysis.

OD measurements were performed in a 96-well plate incubated at 55 °C in the absence of oxygen as previously described [27]. Each well contained 200 μL MTC-6 medium. The plate was shaken for 30 s every 3 min, followed by measuring the optical density at 600 nm.

*Escherichia coli* strains used for cloning were grown aerobically at 37 °C in Lysogeny Broth (LB) [28] medium with either kanamycin (200 μg/mL) or erythromycin (25 μg/mL). For cultivation on solid medium, 15 g/L agarose was added.

All reagents used were from Sigma-Aldrich unless otherwise noted. All solutions were made with water purified using a MilliQ system (Millipore, Billerica, MA, USA)

### Plasmid construction

Plasmids for gene deletion were designed as previously described [29] with either kanamycin or erythromycin resistance cassettes from plasmids pMU433 or pZJ23 flanked by 1.0- to 0.5-kb regions homologous to the 5′ and 3′ regions of the deletion target of interest. Plasmids pZJ13, pZJ15, pZJ16, pZJ17 and pZJ20 were created based on pMU433. The backbone and kanamycin cassettes from plasmid pMU433 were amplified by the primers shown in Table 6. Homologous regions of deletion targets of interest were amplified from wild-type *T. saccharolyticum* (LL1025). Plasmid pZJ23 was created as a new deletion vector by assembling an erythromycin cassette from the ALK2 strain and *E. coli* replication region from plasmid pUC19. Plasmid pZJ25 was based on pZJ23 with homologous regions inserted to allow deletion of *pfl*. The same homologous region on pZJ20 was amplified and cloned on pZJ25.

**Table 6:** Oligonucleotides used in this study

Primer	Target gene	Sequence (5′–3′)
JP75	Kanamycin cassette from pMU433	TAAACCGCTAAGGCATGA
JP76		CTATCTGCATCGTCTTTTC
JP77	pMU433 backbone	AGTTAGGATGTTGGCAGA
JP78		AAAGAGGGCATACAAGGA
JP209	Erythromycin cassette from ALK2	TGCAGGTCGATAAACCCAG
JP210		GAATTCCCTTTAGTAACGTGTAACTTTC
JP211	Replication region from pUC19	CATTAATGAATCGGCCAAC
JP212		CTCGTGATACGCCTATTT
JP143	external to *pforA* cluster	GCTGTGGCAACTTAACAA
JP144		CTCATATCATCCGCTCCT
JP167	external to *pforB* cluster	GTTGTTGTTTTGGCTTAGG
JP168		AGGCTTTCATTCAGTACG
JP169	external to *pforD* cluster	CGTGCCTTTTGACCTTCC
JP170		CTGCTGTCTCGTCCTATT
JP171	external to *pforF* cluster	CCAATATACCACCAGCCA
JP172		GAATTTAGGAAAACCGCCA
JP181	external to *pfl* cluster	ATCCCTCTGTGTCTTTATC
JP182		TGGTTGTGGGTGTTTATG
*recA*-F	qPCR for Tsac_1846 (*recA*)	GAAGCCTTAGTGCGAAGTGG
*recA*-R		GAAGTCCAACATGTGCATCG
*pfor*-F	qPCR for Tsac_0046	ATCAAGCTTGGAATGGGTTG
*pfor*-R		GCTGTTGGAGCCTTTGAGTC
*pfl*-F	qPCR for Tsac_0628	CTATAGCATCGCCTGCTGTG
*pfl*-R		TCGATACCGCCGTTTATAGC
*pfl_ae*-F	qPCR for Tsac_0629	ATTGCCATAACCCTGACACA
*pfl_ae*-R		TAGGCTCTCCACCTGTCAGC

Plasmids were assembled by Gibson Assembly Master Mix (New England Biolabs, Ipswich, MA). The assembled circular plasmids were transformed into *E. coli* DH5α chemical competent cells (New England Biolabs, Ipswich, MA) for propagation. Plasmids were purified by a Qiagen miniprep kit (Qiagen Inc., Germantown, MD, USA).

### Transformation of *T. saccharolyticum*

Plasmids were transformed into naturally competent *T. saccharolyticum* as described before [25, 30]. Mutants were grown and selected on solid medium with kanamycin (200 μg/mL) at 55 °C or with erythromycin (20 μg/mL) at 48 °C in an anaerobic chamber (COY Labs, Grass Lake, MI, USA). Mutant colonies appeared on selection plates after about 3 days. Target gene deletions, with chromosomal integration at both homology regions, were confirmed by PCR with primers external to the target genes (Table 6).

### Preparation of cell-free extracts

*Thermoanaerobacterium saccharolyticum* cells were grown in CTFUD medium in an anaerobic chamber (COY labs, Grass Lake, MI, USA), and harvested in the exponential phase of growth at OD between 0.6 and 0.8. To prepare cell-free extracts, cells were collected by centrifugation at 6,000×*g* for 15 min and washed twice under similar conditions with a deoxygenated buffer containing 100 mM Tris–HCl (pH 7.5) and 5 mM dithiothreitol (DTT). Cells from 50 mL culture were resuspended in 3 mL of the washing buffer. Resuspended cells were lysed by adding 10 μL of 1:100 diluted Ready-Lyse lysozyme solution (Epicentre, Madison, WI, USA) and 2 μL of DNase I solution (Thermo scientific, Waltham, MA, USA) and then incubated at room temperature for 20 min. The concentration of Ready-Lyse lysozyme solution varies from 20 to 40 KU/μL and the DNase I solution is 25 U/μL. The crude lysate was centrifuged at 12,000×*g* for 5 min and the supernatant was collected as cell-free extract. The total amount of protein in the extract was determined by Bradford assay [31], using bovine serum albumin as the standard.

### Enzymes assays

Enzyme activity was assayed in an anaerobic chamber (COY labs, Grass Lake, MI, USA) using an Agilent 8453 spectrophotometer with Peltier temperature control module (part number 89090A) to maintain assay temperature. The reaction volume was 1 mL, in reduced-volume quartz cuvettes (part number 29MES10; Precision Cells Inc., NY, USA) with a 1.0 cm path length. The units for all enzyme activities are expressed as μmol of product · min^−1^ (mg of cell extract protein)^−1^. For each enzyme assay, at least two concentrations of cell extract were used to confirm that the specific activity was proportional to the amount of extract added.

All chemicals and coupling enzymes were purchased from Sigma except for coenzyme A, which was purchased from EMD Millipore (Billerica, MA, USA). All chemical solutions were prepared fresh weekly.

Pyruvate ferredoxin oxidoreductase was assayed by the reduction of methyl viologen, which was monitered at 578 nm,  at 55 °C with minor modifications as described before [32]. An extinction coefficient of *ξ*_578_ = 9.7/mM/cm was used for calculating the activity. The assay mixture contained 100 mM Tris–HCl (pH = 7.5), 5 mM DTT, 2 mM MgCl_2_, 0.4 mM coenzyme A, 0.4 mM thiamine pyrophosphate, 1 mM methyl viologen, cell extract and approximately 0.25 mM sodium dithionite (added until faint blue, A_578_ = 0.05–0.15). The reaction was started by adding 10 mM sodium pyruvate. Activities were expressed as acetyl-CoA production rate.

### Adaptation experiment

Inside the anaerobic chamber, strains were inoculated into polystyrene tubes (Corning, Tewksbury, MA, USA), containing 10 mL MTC-6 medium. The growth of cells in culture was determined by measuring OD_600_. 200 μL of cultures was transferred into tubes with 10 mL fresh medium at the exponential phase of growth as indicated by OD_600nm_ = 0.3.

### RNA isolation, RT-PCR and qPCR for determining transcriptional expression level

3 mL of bacterial culture was pelleted and lysed by digestion with lysozyme (15 mg/mL) and proteinase K (20 mg/mL). RNA was isolated with an RNeasy minikit (Qiagen Inc., Germantown, MD, USA) and digested with TURBO DNase (Life Technologies, Grand Island, NY, USA) to remove contaminating DNA. cDNA was synthesized from 500 ng of RNA using the iScript cDNA synthesis kit (Bio-Rad, Hercules, CA, USA). Quantitative PCR (qPCR) was performed using cDNA with SsoFast EvaGreen Supermix (Bio-Rad, Hercules, CA, USA) at an annealing temperature of 55 °C to determine expression levels of Tsac_0046, Tsac_0628 and Tsac_0629. In each case, expression was normalized to *recA* RNA levels. To confirm removal of contaminating DNA from RNA samples, cDNA was synthesized in the presence and absence of reverse transcriptase followed by qPCR using *recA* primers to ensure only background levels were detected in the samples lacking reverse transcriptase. Standard curves were generated using a synthetic DNA template (gBlock, IDT, Coralville, IA, USA) containing the amplicons. Primers used for qPCR are listed in Table 6.

### Genomic sequencing

Genomic DNA was submitted to the Joint Genome Institute (JGI) for sequencing with an Illumina MiSeq instrument. Paired-end reads were generated, with an average read length of 150 bp and paired distance of 500 bp. Raw data were analyzed using CLC Genomics Workbench, version 7.5 (Qiagen, USA). First reads were mapped to the reference genome (NC_017992). Mapping was improved by two rounds of local realignment. The CLC Probabilistic Variant Detection algorithm was used to determine small mutations (single and multiple nucleotide polymorphisms, short insertions and short deletions). Variants occurring in less than 90 % of the reads and variants that were identical to those of the wild-type strain (i.e., due to errors in the reference sequence) were filtered out. The fraction of the reads containing the mutation is presented in Additional file 3: Table S1.

To determine larger mutations, the CLC InDel and Structural Variant algorithm was run. This tool analyzes unaligned ends of reads and annotates regions where a structural variation may have occurred, which are called breakpoints. Since the read length averaged 150 bp and the minimum mapping fraction was 0.5, a breakpoint can have up to 75 bp of sequence data. The resulting breakpoints were filtered to eliminate those with fewer than ten reads or less than 20 % “not perfectly matched.” The breakpoint sequence was searched with the Basic Local Alignment Search Tool (BLAST) algorithm [33] for similarity to known sequences. Pairs of matching left and right breakpoints were considered evidence for structural variations such as transposon insertions and gene deletions. The fraction of the reads supporting the mutation (left and right breakpoints averaged) is presented in Additional file 3: Table S1.

Unamplified libraries were generated using a modified version of Illumina’s standard protocol. 100 ng of DNA was sheared to 500 bp using a focused ultrasonicator (Covaris). The sheared DNA fragments were size selected using SPRI beads (Beckman Coulter). The selected fragments were then end repaired, A tailed and ligated to Illumina compatible adapters (IDT, Inc) using KAPA-Illumina library creation kit (KAPA biosystems). Libraries were quantified using KAPA Biosystem’s next-generation sequencing library qPCR kit and run on a Roche LightCycler 480 real-time PCR instrument. The quantified libraries were then multiplexed into pools for sequencing. The pools were loaded and sequenced on the Illumina MiSeq sequencing platform utilizing a MiSeq Reagent Kit v2 (300 cycle) following a 2 × 150 indexed run recipe.

### Analytical techniques

Fermentation products: cellobiose, glucose, acetate, lactate, formate, pyruvate, succinate, malate and ethanol were analyzed by a Waters (Milford, MA) high-pressure liquid chromatography (HPLC) system with an Aminex HPX-87H column (Bio-Rad, Hercules, CA, USA). The column was eluted at 60 °C with 0.25 g/L H_2_SO_4_ at a flow rate of 0.6 mL/min. Cellobiose, glucose, acetate, lactate, formate, succinate, malate and ethanol were detected by a Waters 410 refractive-index detector and pyruvate was detected by a Waters 2487 UV detector. Sample collection and processing were as reported previously [34].

Carbon from cell pellets was determined by elemental analysis with a TOC-V CPH and TNM-I analyzer (Shimadzu, Kyoto, Japan) operated by TOC-Control V software. Fermentation samples were prepared as described with small modifications [35]. A 1 mL sample was centrifuged to remove the supernatant at 21,130*g* for 5 min at room temperature. The cell pellet was washed twice with MilliQ water. After washing, the pellet was resuspended in a TOCN 25 mL glass vial containing 19.5 mL MilliQ water. The vials were then analyzed by the TOC-V CPH and TNM-I analyzer.

Hydrogen was determined by gas chromatography using a Model 310 SRI Instruments (Torrence, CA, USA) gas chromatograph with a HayeSep D packed column using a thermal conductivity detector and nitrogen carrier gas. The nitrogen flow rate was 8.2 mL/min.

Carbon balances were determined according to the following equations, accounting for carbon dioxide and formate through the stoichiometric relationship of its production to levels of acetate, ethanol, malate and succinate [25]. The overall carbon balance is as follows:

$$C_{t} = 12{\text{CB}} + 6{\text{G}} + 3{\text{L}} + 3{\text{A}} + 3{\text{E}} + 3{\text{P}} + 3{\text{M}} + 3{\text{S}} + 1{\text{Pe}},$$

where *C*_t_ is the total carbon, CB the cellobiose, G the glucose, L the lactate, E the ethanol, P the pyruvate, M the malate, S the succinate, Pe the pellet and

$$C_{\text{R}} = \frac{{C_{\text{tf}} }}{{C_{\text{t0}} }} \times 100\,\,\% ,$$

where *C*_R_ is the carbon recovery, *C*_t0_ the total carbon at the initial time, and *C*_tf_ the total carbon at the final time. Electron recoveries were calculated in a similar way, with the following numbers of available electrons per mole of compound: per mole 48 for cellobiose, 24 for glucose, 8 for acetate, 12 for ethanol, 12 for lactate, 14 for succinate, 10 for pyruvate, 12 for malate, 2 for hydrogen and 2 for formate. The electrons contained in the cell pellet was estimated with a general empirical formula for cell composition (CH_2_N_0.25_O_0.5_); therefore, the available electrons per mole cell carbon was assumed to be 4.75 per mole. The calculation follows the equations below:

$$E_{t} = 48{\text{CB}} + 24{\text{G}} + 12{\text{L}} + 8{\text{A}} + 12{\text{E}} + 14{\text{S}} + 10{\text{P}} + 12{\text{M}} + 2{\text{H}} + 2{\text{F}} + 4.75{\text{Pe,}}$$

$$E_{\text{R}} = \frac{{E_{\text{tf}} }}{{E_{\text{t0}} }} \times 100\,\,\% ,$$

where *E*_t_ is the total electrons, *E*_R_ the electron recovery, F the formate and H the hydrogen; other abbreviations are the same as shown above.

## Additional files

Additional file 1:
**Figure S1.** Growth curves of Δpfor strains in MTC-6 medium with 4.5 g/L yeast extract. Black plus represent wild type strain (LL1025), black cyan circle represent Δpfor-1, green magenta cross represent Δpfor-2, blue diamond represent adapted Δpfor-1, red star represent adapted Δpfor-2. Additional file 2:
**Figure S2.** Growth curves of Δ*pfl* strains in MTC-6 medium with and without yeast extract. Lines represent growth curves of wild type (black), Δ*pfl*-1(red), Δ*pfl*-2 (blue) in MTC-6 medium with 4.5 g/L yeast extract. Circles represent growth curves of wild type (black), Δ*pfl*-1(red), Δ*pfl*-2 (blue) in MTC-6 medium without yeast extract. Additional file 3:
**Table S1.** Mutations found in genomic analysis.

## Abbreviations

CBP: consolidated bioprocessing
PFOR: pyruvate ferredoxin oxidoreductase
PFL: pyruvate formate-lyase
PDH: pyruvate dehydrogenase
PDC: pyruvate decarboxylase
THF: tetrahydrofolate

## References

[1] Lee Y-E, Jain MK, Lee C, Zeikus JG (1993). Taxonomic distinction of saccharolytic thermophilic anaerobes: description of *Thermoanaerobacterium xylanolyticum* gen. nov., sp. nov., and *Thermoanaerobacterium saccharolyticum* gen. nov., sp. nov.; reclassification of *Thermoanaerobium brockii*, *Clostridium thermosulfurogenes*, and* Clostridium thermohydrosulfuricum* E100-69 as *Thermoanaerobacter brockii* comb. nov., *Thermoanaerobacterium thermosulfurigenes* comb. nov., and *Thermoanaerobacter thermohydrosulfuricus* comb. nov., Respectively; and Transfer of *Clostridium thermohydrosulfuricum* 39E to* Thermoanaerobacter ethanolicus*. Int J Syst Bacteriol.

[2] Shaw AJ, Podkaminer K, Desai S, Bardsley J, Rogers S, Thorne P (2008). Metabolic engineering of a thermophilic bacterium to produce ethanol at high yield. Proc Natl Acad Sci USA.

[3] Shaw AJ, Covalla SF, Miller BB, Firliet BT, Hogsett DA, Herring CD (2012). Urease expression in a *Thermoanaerobacterium saccharolyticum* ethanologen allows high titer ethanol production. Metab Eng.

[4] Herring CD, Kenealy WR, Shaw AJ, Raman B, Tschaplinski TJ, Brown SD (2012). Final report on development of *Thermoanaerobacterium saccharolyticum* for the conversion of lignocellulose to ethanol.

[5] Lynd LR, Weimer PJ, van Zyl WH, Pretorius IS (2002). Microbial cellulose utilization: fundamentals and biotechnology. Microbiol Mol Biol Rev.

[6] Lynd LR, van Zyl WH, McBride JE, Laser M (2005). Consolidated bioprocessing of cellulosic biomass: an update. Curr Opin Biotechnol.

[7] Olson DG, Sparling R, Lynd LR (2015). Ethanol production by engineered thermophiles. Curr Opin Biotechnol.

[8] Lo J, Zheng T, Hon S, Olson DG, Lynd LR (2015) The bifunctional alcohol and aldehyde dehydrogenase gene, adhE, is necessary for ethanol production in *Clostridium thermocellum* and *Thermoanaerobacterium saccharolyticum*. J Bacteriol 197:JB.02450–14 10.1128/JB.02450-14 PMC4372742 25666131

[9] Shaw AJ, Jenney FE, Adams MWW, Lynd LR (2008). End-product pathways in the xylose fermenting bacterium, *Thermoanaerobacterium saccharolyticum*. Enzyme Microb Technol.

[10] Kanehisa M, Goto S (2000). KEGG : Kyoto encyclopedia of genes and genomes. Nucleic Acids Res.

[11] Kanehisa M, Goto S, Sato Y, Kawashima M, Furumichi M, Tanabe M (2014). Data, information, knowledge and principle: back to metabolism in KEGG. Nucleic Acids Res.

[12] Chabriere E, Cavazza C, Contreras-Martel C, Fontecilla-Camps JC (2011) Pyruvate–ferredoxin oxidoreductase. In: Encyclopedia of inorganic and bioinorganic chemistry. Wiley, pp 1–13. http://onlinelibrary.wiley.com/doi/10.1002/9781119951438.eibc0647/abstract

[13] Currie DH, Guss AM, Herring CD, Giannone RJ, Johnson CM, Lankford PK (2014). Profile of secreted hydrolases, associated proteins, and SlpA in *Thermoanaerobacterium saccharolyticum* during the degradation of hemicellulose. Appl Environ Microbiol.

[14] Lo J, Zheng T, Olson DG, Ruppertsberger N, Tripathi SA, Guss AM et al (2015) Deletion of *nfnAB* in *Thermoanaerobacterium saccharolyticum* and its effect on metabolism. J Bacteriol. doi:10.1128/JB.00347-15 10.1128/JB.00347-15 PMC4542167 26124241

[15] Zhou J, Olson DG, Argyros D, Deng Y, van Gulik W, van Dijken J (2013). Atypical glycolysis in *Clostridium thermocellum*. Appl Environ Microbiol.

[16] Ma K, Hutchins A, Sung SJ, Adams MW (1997). Pyruvate ferredoxin oxidoreductase from the hyperthermophilic archaeon, *Pyrococcus furiosus*, functions as a CoA-dependent pyruvate decarboxylase. Proc Natl Acad Sci USA.

[17] Eram MS, Oduaran E, Ma K (2014). The bifunctional pyruvate decarboxylase/pyruvate ferredoxin oxidoreductase from *Thermococcus guaymasensis*. Archaea.

[18] Basen M, Schut GJ, Nguyen DM, Lipscomb GL, Benn RA, Prybol CJ (2014). Single gene insertion drives bioalcohol production by a thermophilic archaeon. Proc Natl Acad Sci USA.

[19] Van Den Ban ECD, Willemen HM, Wassink H, Laane C, Haaker H (1999). Bioreduction of carboxylic acids by *Pyrococcus furiosus* in batch cultures. Enzyme Microb Technol.

[20] Thauer RK, Kirchniawy FH, Jungermann KA (1972). Properties and function of the pyruvate-formate-lyase reaction in Clostridiae. Eur J Biochem.

[21] Zeikus J (1983). Metabolism of one-carbon compounds by chemotrophic anaerobes. Adv Microb Physiol.

[22] Amador-Noguez D, Feng X, Fan J, Roquet N, Rabitz H, Rabinowitz JD (2010). Systems-level metabolic flux profiling elucidates a complete, bifurcated tricarboxylic acid cycle in *Clostridium acetobutylicum*. J Bacteriol.

[23] Cronan JE, Zhao X, Jiang Y (2005). Function, attachment and synthesis of lipoic acid in *Escherichia coli*. Adv Microb Physiol.

[24] Wigley DB, Gamblin SJ, Turkenburg JP, Dodson EJ, Piontek K, Muirhead H (1992). Structure of a ternary complex of an allosteric lactate dehydrogenase from *Bacillus stearothermophilus* at 2.5 A resolution. J Mol Biol.

[25] Shaw AJ, Hogsett DA, Lynd LR (2009). Identification of the [FeFe]-hydrogenase responsible for hydrogen generation in *Thermoanaerobacterium saccharolyticum* and demonstration of increased ethanol yield via hydrogenase knockout. J Bacteriol.

[26] Hogsett DA (1995). Cellulose hydrolysis and fermentation by *Clostridium thermocellum* for the production of ethanol.

[27] Olson DG, Lynd LR (2012). Computational design and characterization of a temperature-sensitive plasmid replicon for gram positive thermophiles. J Biol Eng.

[28] Bertani G (1951). Studies on lysogenesis. I. The mode of phage liberation by lysogenic *Escherichia coli*. J Bacteriol.

[29] Desai SG, Guerinot ML, Lynd LR (2004). Cloning of l-lactate dehydrogenase and elimination of lactic acid production via gene knockout in *Thermoanaerobacterium saccharolyticum* JW/SL-YS485. Appl Microbiol Biotechnol.

[30] Shaw AJ, Hogsett DA, Lynd LR (2010). Natural competence in *Thermoanaerobacter* and *Thermoanaerobacterium* species. Appl Environ Microbiol.

[31] Kruger N (1994). The Bradford method for protein quantitation. Methods Mol Biol.

[32] Ma K, Adams MW (2001). Ferredoxin: NADP oxidoreductase from *Pyrococcus furiosus*. Methods Enzymol.

[33] Altschul SF, Gish W, Miller W, Myers EW, Lipman DJ (1990). Basic local alignment search tool. J Mol Biol.

[34] Zhang YP, Lynd LR (2005). Regulation of cellulase synthesis in batch and continuous cultures of *Clostridium thermocellum*. J Bacteriol.

[35] Van der Veen D, Lo J, Brown SD, Johnson CM, Tschaplinski TJ, Martin M (2013). Characterization of *Clostridium thermocellum* strains with disrupted fermentation end-product pathways. J Ind Microbiol Biotechnol.

