# Supplementary figures and images for: Physiological roles of pyruvate ferredoxin oxidoreductase and pyruvate formate-lyase in Thermoanaerobacterium saccharolyticum JW/SL-YS485
Source: Biotechnol Biofuels. 2015 Sep 15;8:138. doi: 10.1186/s13068-015-0304-1 (PMC4570089; doi:10.1186/s13068-015-0304-1)

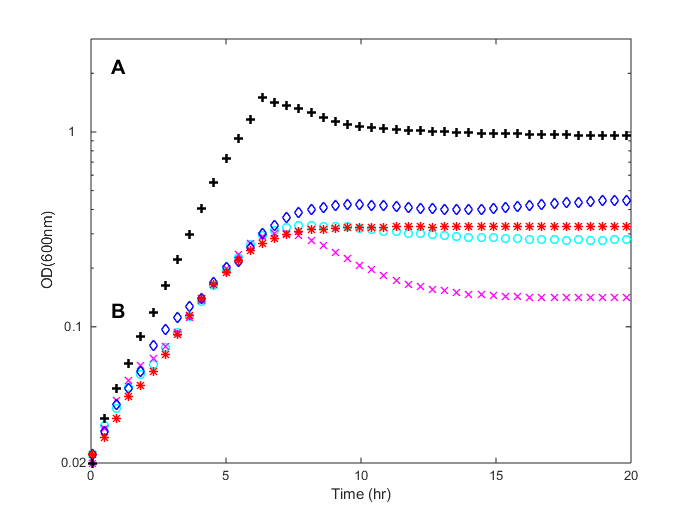

Supplement: Additional file 1: — Figure S1. Growth curves of Δpfor strains in MTC-6 medium with 4.5 g/L yeast extract. Black plus represent wild type strain (LL1025), black cyan circle represent Δpfor-1, green magenta cross represent Δpfor-2, blue diamond represent adapted Δpfor-1, red star represent adapted Δpfor-2. [file 13068_2015_304_MOESM1_ESM.png]

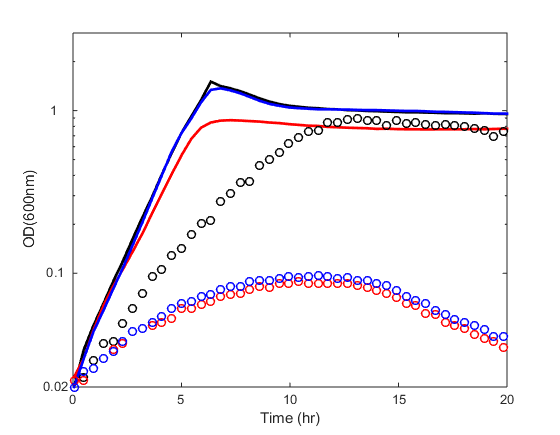

Supplement: Additional file 2: — Figure S2. Growth curves of Δpfl strains in MTC-6 medium with and without yeast extract. Lines represent growth curves of wild type (black), Δpfl-1(red), Δpfl-2 (blue) in MTC-6 medium with 4.5 g/L yeast extract. Circles represent growth curves of wild type (black), Δpfl-1(red), Δpfl-2 (blue) in MTC-6 medium without yeast extract. [file 13068_2015_304_MOESM2_ESM.png]
