# Supplementary material for: Physiological roles of pyruvate ferredoxin oxidoreductase and pyruvate formate-lyase in Thermoanaerobacterium saccharolyticum JW/SL-YS485
Source: Biotechnol Biofuels. 2015 Sep 15;8:138. doi: 10.1186/s13068-015-0304-1 (PMC4570089; doi:10.1186/s13068-015-0304-1)
Supplement: Additional file 3: — Table S1. Mutations found in genomic analysis. [file 13068_2015_304_MOESM3_ESM.pdf]

Table S1. Mutations found in genomic analysis*

	Locus	Description	Start	Type	Amino acid change			lineage 1			lineage 2		lineage 3	
						LL1025	LL1049	LL1139	LL1141	LL1178	LL1140	LL1142	LL1170	LL1164
Targeted gene deletions	Tsac_0046	Pyruvate-ferredoxin oxidoreductase	47169	pfor deletion marked with pta-ack kan cassette	n/a			0.9	1.0	0.9	1.0	1.0		
	Tsac_0179	Lactate dehydrogenase	190602	"Clean" ldh deletion	n/a		1.0							
	Tsac_0628	Pyruvate formate lyase	665772	pfl deletion marked with kan	n/a								1.0	1.0
	Tsac_0628	Pyruvate formate lyase	665772	pfl deletion marked with erm	n/a					1.0				
	Tsac_0795	SNF2-related protein	832219	*C. thermocellum* urease operon insertion	n/a		1.0							
	Tsac_1472	Putative uncharacterized protein;	1553610	"Clean" deletion of EPS operon	n/a		1.0							
	Tsac_1704	Orotidine 5'-phosphate decarboxylase	1774294	"Clean" pyrF deletion	n/a									
	Tsac_1744	Phosphate acetyltransferase	1814202	"Clean" pta-ack deletion	n/a		1.0	0.3	0.4		0.2	0.2		
	Tsac_2828	hypothetical protein	21	MNV	Lys104del/insLysGlu		1.0							
	Tsac_0076	Amino acid permease-associated region;	76166	SNV	Thr250Lys					1.0				
	Tsac_0076	Amino acid permease-associated region;	76511	SNV	Gly135Glu				1.0					
	Tsac_0079	Putative uncharacterized protein;	79429	Deletion	Asn86fs		1.0							
	Tsac_0144	4-deoxy-L-threo-5-hexosulose-uronate ketol-isomerase;	150053	SNV	none, upstream of CDS		1.0							
	Tsac_0153	Mannitol dehydrogenase domain;	164232	Insertion	Arg208fs									
	Tsac_0163	Mannitol dehydrogenase domain;	177035	SNV	Phe104Val		1.0							
	Tsac_0176	FAD-dependent pyridine nucleotide-disulfide oxidoreductase;	189536	Tsac_2745 (transposon?) insertion	n/a					0.9				
	Tsac_0179	L-lactate dehydrogenase 1;	191093	Insertion	Asn185Ser						1.0	1.0		
	Tsac_0179	L-lactate dehydrogenase 1;	191671	SNV	none, upstream of CDS					1.0				
	Tsac_0377	Amino acid-binding ACT domain protein;	408901	MNV	Arg11fs				1.0	1.0				
	Tsac_0390	CRISPR-associated HD domain protein;	424392	101 bp deletion	n/a		1.0							
	Tsac_0416	Acetaldehyde/alcohol dehydrogenase;	448858	SNV	Gly544Asp		1.0							
	Tsac_0416	Acetaldehyde/alcohol dehydrogenase;	449032	SNV	Ser602Leu									
	Tsac_0535	Cyanophycin synthetase;	559915	SNV	Gly257Asp		1.0							
	Tsac_0628	Pyruvate formate lyase;	665702	SNV	none, upstream of CDS				1.0					
	Tsac_0727	Putative uncharacterized protein;	762092	SNV	Arg113*				1.0	1.0				
	Tsac_0838	Putative uncharacterized protein;	884294	Deletion	Asp99fs		1.0							
	Tsac_0948	Spore germination protein;	993551	SNV	Phe361Tyr		1.0							
	Tsac_0996	Translation elongation factor Tu;	1042678	SNV	none, upstream of CDS					1.0				
	Tsac_1054	Thioredoxin reductase;	1089642	SNV	none, upstream of CDS		1.0							
	Tsac_1057	ATPase associated with various cellular activities AAA_3;	1092445	SNV	no change, synonymous									
	Tsac_1241	PDZ/DHR/GLGF domain protein;	1284739	SNV	Ser183*				0.9					
	Tsac_1263	PTS system transcriptional activator;	1313539	Deletion	Phe370_Phe371delinsPhe			0.7						
	Tsac_1263	PTS system transcriptional activator;	1314647	SNV	Val2Leu				0.9					
	Tsac_1296	UDP-glucose 4-epimerase;	1356848	SNV	Glu105Gly		1.0							
	Tsac_1304	Putative uncharacterized protein;	1364211	SNV	no change, synonymous									1.0
	Tsac_1314	Amino acid permease-associated region;	1373453	Tsac_2745 (transposon) insertion	n/a				0.9	1.0				
	Tsac_1327	Putative uncharacterized protein;	1386790	MNV	Asn22fs				1.0	1.0				
	Tsac_1328	Putative uncharacterized protein;	1387303	Deletion	Ile68fs					1.0				
	Tsac_1419	ATP synthase F0, A subunit;	1491395	SNV	Ala187Asp		1.0							
	Tsac_1419	ATP synthase F0, A subunit;	1491415	SNV	Asp194Tyr									
	Tsac_1551	HfsB;	1627813	Deletion	Glu145fs		1.0							
	Tsac_1553	Ferredoxin hydrogenase;	1630591	SNV	Arg107Ser		1.0							
	Tsac_1553	Ferredoxin hydrogenase;	1631586	SNV	Cys439Tyr									1.0
	Tsac_1712	Diaminopimelate epimerase;	1787698	SNV	Pro161Thr		1.0							
	Tsac_1726	Serine/threonine protein kinase with PASTA sensor(S);	1799619	Deletion	Lys4fs				0.9					
	Tsac_1726	Serine/threonine protein kinase with PASTA sensor(S);	1800908	Insertion	Leu433fs					1.0				
	Tsac_1726	Serine/threonine protein kinase with PASTA sensor(S);	1800969	Deletion	Tyr454fs		1.0							
	Tsac_1745	Acetate kinase 2;	1815452	Insertion	Tyr15fs					0.7				
	Tsac_1746	Hypothetical protein (also downstream region of pta-ack cassette)	1816708	SNV	His11Arg			0.7						
	Tsac_1777	GTP-sensing pleiotropic transcriptional repressor CodY;	1844730	Insertion	Ile3fs				0.7					
	Tsac_1782	Flagellar motor switch protein FliG;	1848944	Deletion	Glu180fs		1.0							
	Tsac_1961	RNA polymerase, sigma 28 subunit, FliA/WhiG subfamily;	2009358	TSHA-485A phage insertion	n/a	0.9		0.9	0.9	0.9	0.7	0.9	0.9	0.9
	Tsac_2087	Iron-containing alcohol dehydrogenase;	2133733	Deletion	Asp71fs							0.8		
	Tsac_2100	YheO-like domain-containing protein;	2145983	Insertion	Thr71fs			0.8						
	Tsac_2119	Penicillin-binding protein 2;	2160615	SNV	Gly488Glu		1.0							
	Tsac_2196	Phenylalanyl-tRNA synthetase, alpha subunit;	2239931	SNV	no change, synonymous		1.0							
	Tsac_2229	Inner-membrane translocator;	2273080	SNV	Ser283Pro		1.0							
	Tsac_2390	Putative uncharacterized protein;	2460517	SNV	Leu311Phe		1.0							
	Tsac_2476	RNA polymerase, sigma 70 subunit, RpoD subfamily;	2526425	SNV	no change, synonymous		1.0							
	Tsac_2507	PTS system transcriptional activator;	2555785	Insertion	Gln840*		1.0							
	Tsac_2507	PTS system transcriptional activator;	2557246	SNV	Ala353fs				0.8					
	Tsac_2564	Phosphotransferase system PTS lactose/cellobiose-specific IIA subunit;	2618782	Tsac_2745 (transposon) insertion	n/a		1.0							
	Tsac_2602	Extracellular ligand-binding receptor;	2652049	1,969 bp deletion	n/a					1.0				

* values indicate fraction of reads with mutation. In some strains (LL1139, LL1140, LL1141 and LL1142), low values for the mutation fraction in the pta gene (Tsac_1744) are the result of the presence of two copies of the pta-ack cassette at different loci on the chromosome.
